# Estimating prevalence and identifying predictors of zero-dose pentavalent and never-immunized children under two years of age in Kashmore and Sujawal Districts of Sindh, Pakistan: An analysis of household survey data

**DOI:** 10.1371/journal.pone.0330281

**Published:** 2025-08-26

**Authors:** Danya Arif Siddiqi, Manaksha Memon, Sundus Iftikhar, Muhammad Siddique, Vijay Kumar Dharma, Ahsan Ahmad, Nauman Safdar, Mubarak Taighoon Shah, Hamidreza Setayesh, Irshad Ali Sodhar, Farrukh Raza Malik, Subhash Chandir

**Affiliations:** 1 IRD Global, Hong Leong Building, Singapore, Singapore; 2 London School of Hygiene & Tropical Medicine, London, United Kingdom; 3 IRD Pakistan, Korangi Creek, Karachi, Pakistan; 4 Prime Consulting, Islamabad, Pakistan; 5 Bill & Melinda Gates Foundation, Seattle, Washington, United States of America; 6 Department of Health, Government of Sindh, Karachi, Pakistan; 7 Establishment Division, Government of Pakistan, Pakistan; Marie Stopes International, PAKISTAN

## Abstract

**Introduction:**

Despite intensified global efforts to enhance immunization coverage, one in five children continue to miss out on life-saving vaccines, leaving them vulnerable to a range of vaccine-preventable diseases. In 2022, 14.3 million children failed to receive even a single dose of the pentavalent vaccine (Penta-1) by their first birthday, classified as “zero-dose penta”. Additionally, some children have not received any vaccinations at all and have had no contact with healthcare services—these are referred to as “never-immunized” children. Collectively, both groups—zero-dose penta and never-immunized children—are termed “true zero-dose” to emphasize the critical need for targeted interventions that ensure no child is left behind in immunization efforts.

**Methods:**

We conducted a household (HH) survey from August 10 to December 19, 2022, in Kashmore and Sujawal, two districts in Sindh, Pakistan, with low immunization coverage. The survey targeted children aged 12−23 months who had not received the Penta-1 vaccine by their first birthday. Our study aimed to determine the community-based prevalence of zero-dose penta and never-immunized children, compare their sociodemographic characteristics and immunization histories, and identify predictors of these outcomes.

**Results:**

Of the 2,091 children surveyed, 497 (23.8%) were zero-dose penta, and 587 (28.1%) were never-immunized. Together, these groups constitute 51.9% of the survey population, referred to as ‘true zero-dose’. The remaining 1,007 (48.1%) were either fully or partially immunized. Multivariate analysis indicated that absence of antenatal care (ANC) significantly increased the risk of children being classified as zero-dose penta (RRR = 1.68; 95% CI: 1.04–2.72; p < 0.035) and never-immunized (RRR = 2.07; 95% CI: 1.25–3.45; p < 0.005). Furthermore, the absence of Lady Health Worker (LHW) visits significantly increased the risk of children being classified as zero-dose penta (RRR = 2.55; 95% CI: 1.26–5.16; p < 0.009), and the absence of vaccinator visits significantly increased the risk of being never-immunized (RRR = 4.44; 95% CI: 2.68–7.36; p < 0.001).

**Conclusion:**

Despite global efforts for achieving universal immunization, half of the surveyed children remained true zero-dose, highlighting significant gaps in the ability of immunization programs to reach underserved communities. To address this issue, it is essential to enhance ANC coverage and leverage frontline health workers (FHWs) to identify and engage with clusters of zero-dose children effectively. These measures will ensure that no child is left behind, advancing health equity and safeguarding future generations.

## Introduction

Despite intensified global efforts to enhance immunization coverage, one in five children continue to miss out on life-saving vaccines, leaving them vulnerable to a range of vaccine-preventable diseases [1]. In 2022, 14.3 million children failed to receive even a single dose of the pentavalent vaccine (Penta-1) by their first birthday, classified as “zero-dose penta” [2]. Additionally, some children have not received any vaccinations at all and have had no contact with healthcare services—these are referred to as “never-immunized” children [3]. Collectively, both groups—zero-dose penta and never-immunized children—are termed “true zero-dose” to emphasize the critical need for targeted interventions that ensure no child is left behind in immunization efforts. These infants are disproportionately found in low-resource settings, including urban slums, remote rural areas, and conflict-affected settings, characterized by limited access to basic health services, inadequate sanitation facilities and poverty [4]. Almost two-thirds of these children in Gavi-supported nations reside in households (HHs) with incomes below the international poverty line of US$ 1.90 per day [5]. The distinct category of never-immunized children presents additional challenges, as their complete lack of interaction with healthcare systems makes them difficult to identify and track, consequently placing them at a heightened risk of contracting vaccine-preventable diseases [6]. While all never-immunized children are inherently zero-dose penta, the converse is not true—some zero-dose penta children might have received other vaccines but missed the Penta-1 dose. The Immunization Agenda 2030 aims to halve the number of zero-dose penta children by 2030 and promises to leave no one behind with immunization by sustainably integrating never-immunized children into standard vaccination programs [7].

In Pakistan, concerted efforts have substantially reduced the number of zero-dose penta children by 29%, yet the country still ranks ninth globally in terms of the number of zero-dose penta children (431,000) [2]. This is compounded by the challenge that Pakistan ranks third globally for having the most never-immunized children [8]. The effectiveness of current interventions is often limited by the lack of precise data to accurately assess the extent of zero-dose penta and never-immunized children. A study in the Sindh province of Pakistan found that one in every ten children was zero-dose penta, but this figure might be underrepresented due to the study’s methodology, which relied on data from children enrolled in the provincial electronic immunization registry only, thereby excluding those who have never interacted with the healthcare system [9]. The gaps in data significantly hinder efforts to ensure equitable immunization access, leading to clusters of unimmunized children. Beyond immediate health risks, high rates of zero-dose and never-immunized children have broader implications. Unvaccinated children face increased risks of malnutrition, stunted growth, and cognitive impairments, affecting their education and long-term productivity [10]. The healthcare system also bears the burden, as unvaccinated children require more intensive medical care, straining limited resources. Economically, frequent outbreaks increase healthcare costs, reduce productivity, and slow national development [11]. Cultural beliefs and social norms also play a significant role in vaccine hesitancy [12]. Misinformation, religious misconceptions, and distrust in healthcare providers often discourage parents from immunizing their children [8]. In some communities, patriarchal structures limit mothers’ decision-making power regarding healthcare, further restricting vaccine uptake [13]. Additionally, past experiences with coercive vaccination campaigns have fueled skepticism, making engaging with local leaders and building trust through culturally sensitive communication strategies crucial [14].

To ensure no child is left behind in receiving immunizations, it is essential for stakeholders to accurately assess the prevalence of zero-dose penta and never-immunized children, collectively referred to as ‘true zero-dose’, at a microgeographic level. A deeper understanding of the specific characteristics of these children and the factors contributing to their exclusion from immunization programs is crucial. Gaining these insights is vital for developing context-specific strategies and implementing targeted interventions, enabling effective reach to unimmunized children who are often concentrated in overlooked or underserved communities.

We aimed to assess the community-based prevalence of zero-dose penta and never-immunized children aged 12–23 months through a door-to-door HH survey in two low-coverage districts of Sindh Province, Pakistan. Furthermore, our analysis identified socioeconomic risk factors associated with zero-dose penta and never-immunized children.

## Methods

### Site and population

Sindh Province, with a population of 55.3 million and a density of 392.8 individuals per square kilometer [15], has an annual birth cohort of 1.9 million [16]. The province’s poverty index, a composite measure of multidimensional poverty, is 0.28, meaning that, on average, individuals experience 28% of the maximum possible deprivations across various indicators. This index accounts for factors such as education, health, and living standards, making it a relevant measure for assessing disparities in healthcare access, including immunization coverage. Notably, district-level variations range from 0.02 to 0.50, indicating substantial inequities, with some districts experiencing deprivation levels as high as 50% [17]. Pre-COVID-19, fixed immunization centres were responsible for 60% of all provincial immunizations, with the remaining vaccinations provided through routine outreach [18]. However, post-pandemic, routine outreach, defined as immunization sessions at non-fixed sites achievable within a day, accounts for nearly 60% of immunizations, complemented by enhanced outreach that extends services beyond conventional boundaries to cover wider geographic areas [19].

Geographically, Sindh province is segmented into six divisions and subdivided into 30 districts and 1,130 Union Councils (UCs) [20]. Districts Kashmore and Sujawal reported the lowest Penta-1 coverage, falling below the 10th percentile, at 50.0% and 48.1%, respectively (S1 Table) [21]. Kashmore, in the province’s north, has a population of 1.3 million, with a 45,000 annual birth cohort [16]. Approximately 77% of the district’s population resides in rural areas, and the literacy rate is 31% [16]. The district comprises three towns and 37 UCs [20] and is serviced by 43 immunization clinics and 84 vaccinators. Sujawal district, northeast of Sindh, has a population of 0.91 million and an annual birth cohort of 32,000 [16]. It is predominantly characterized by its rural setting where 89% of the district’s population resides and reports a literacy rate of 25% [16]. The district encompasses five towns and 26 UCs [20], with immunization services facilitated by 55 vaccinators operating across 32 immunization clinics. Both Kashmore and Sujawal districts face significant developmental challenges. Kashmore, strategically located at the intersection of three provinces, struggles with socio-political instability, geographical barriers, and weak law enforcement, resulting in high poverty, crime, and limited access to education and employment. Similarly, Sujawal, despite its historical significance and location along the Indus River, suffers from inefficient governance, inadequate infrastructure, and a fragile agricultural sector, further exacerbated by poor access to education and healthcare. Given these structural barriers, along with low Penta-1 coverage rates, both districts are high-risk areas for zero-dose children, making them relevant for the purpose of our research [22,23]. Furthermore, we have included slum areas in these districts in our study, identified based on the classification by Sindh Katchi Abadis Authority (SKAA). According to the Sindh Katchi Abadis Act, 1987, an area can be declared a Katchi Abadi (slum) if it meets specific conditions, including land ownership considerations, government approvals, and public utility exemptions. Once declared, a slum falls under SKAA jurisdiction, subject to these conditions [24]. SKAA has identified 17 and 30 slums in Kashmore and Sujawal districts, respectively [18]. Beyond these criteria, these areas are characteristic of the conventional definition of slums, “a contiguous settlement where the inhabitants are characterized as having inadequate housing and basic services. Slums are often not recognized and addressed by the public authorities as an integral or equal part of the city” [25].

Vaccination Schedule

**Table:** 

Visits	Child age	Vaccines
First visit	At birth	1) BCG 2) OPV-0 3) Hepatitis B-0
Second visit	6 weeks	1) Penta-1 2) OPV-1 3) PCV-1 4) Rota-1
Third visit	10 weeks	1) Penta-2 2) OPV-2 3) PCV-2 4) Rota-2
Fourth visit	14 weeks	1) Penta-3 2) OPV-3 3) PCV-3 4) IPV
Fifth visit	9 months	1) Measles-1 2) IPV-2 3) TCV
Sixth visit	15 months	1) Measles-2

Additions to the Expanded Programme on Immunization schedule include TCV on January 1, 2020, a second IPV dose on May 3, 2021, and the rubella vaccine on November 15, 2021 [26].

### Study design and procedure

We conducted a cross-sectional, door-to-door HH survey in two districts with low Penta-1 coverage, Kashmore and Sujawal, over a period from August 10 to December 19, 2022. We visited 6,395 HHs in two districts across 9 UCs (Kashmore:5; Sujawal:4), and included 2,094 children aged 12–23 months. A three-stage cluster sampling technique was utilized. In the first stage, districts were sorted in ascending order based on Penta-1 coverage (based on coverage rates reported in a third-party verification immunization survey) [21]. Two districts with the lowest pentavalent 1 coverage, Kashmore (50%) and Sujawal (48.1%), were selected from the four districts below the 10th percentile (S1 Table). In the second stage, union councils (UCs) within each selected district were sorted in ascending order based on Penta-1 coverage (based on coverage rates in the Provincial Electronic Immunization Registry) (S2 Table). From each district, one-third of the UCs with the lowest Penta-1 coverage were selected (Kashmore: 11 out of 33 UCs; Sujawal: 8 out of 25 UCs). Subsequently, half of these selected UCs were chosen via simple random sampling using STATA’s rand command (Kashmore: 5 out of 11 UCs; Sujawal: 4 out of 8 UCs). In the third stage, equal numbers of households with zero-dose children aged 12–23 months were selected from each sampled UC (Kashmore: 82 households per UC; Sujawal: 91 households per UC).

We initiated household sampling from the functional immunization center of each selected UC. A spin of a pen on the ground was performed, and the enumeration teams moved in the direction the pen indicated when it came to a still position. The enumeration teams then identified the first household and sampled the first residential structure on the right side within the village. Subsequently, we selected the next household with an interval of five households, continuing until the required sample size was achieved within each given location. We selected households with children aged 12–23 months who were both vaccinated and non-vaccinated with Penta-1. If the selected household was not eligible, i.e., did not contain a child aged 12–23 months, the next door was selected and assessed for eligibility. In cases of two or more eligible children in the same household (e.g., more than one child of the same mother in the eligible age group, or in an extended family situation, etc.), all eligible children were enrolled. If there were two children aged 12–23 months, one vaccinated and the other non-vaccinated with Penta-1, then both were selected. If there were more than one functional immunization centers in each UC, we selected the center located nearest to the geographical center of the UC.

The survey was conducted by trained enumerators through face-to-face interviews using a semi-structured questionnaire after seeking consent. Data on sociodemographic information of the HH, caregiver and child along with the immunization history of the child was collected.

### Sample size/Power calculation

The sample size was calculated using WHO’s Sample Size Determination in Health Studies Software V2.0.21, targeting a 95% confidence level and a 10% error margin around the zero-dose penta children prevalence estimates of 50% in Kashmore and 52% in Sujawal. Populations of Kashmore and Sujawal were 1,090,336 and 779,062 [16], respectively, with relative weights of 58% for Kashmore and 42% for Sujawal, derived from their population proportions. Adjusting for a design effect of 2 and an anticipated non-response rate of 28%, the final sample size aimed for 909 zero-dose penta children: 527 from Kashmore and 382 from Sujawal. We estimated 6,363 HHs (3,710 HHs in Kashmore and 2,653 HHs in Sujawal) to be visited to reach this target (S3 Table).

### Inclusion criteria

Caregivers of children aged 12–23 months who were living in the sampled households for a period of six months or more were eligible for inclusion in this study.

### Exclusion criteria

Caregivers of children aged less than 12 months or above 23 months and living for a period of less than six months in the sampled households were excluded from the study.

### Ethics

This study was approved by the Institutional Review Board of Interactive Research and Development under IRD_IRB_2022_01_002. The IRB is registered with the U.S. Department of Health and Human Services Office for Human Research Protections with registration number IRB 404 00005148.

A detailed verbal consent process was developed and translated into the local language to ensure accessibility. Enumerators explained the study objectives, procedures, benefits, and risks to each caregiver. Participants were provided with the opportunity to read the consent form themselves, and if they were unable to do so, a relative or friend was asked to read it aloud on their behalf. Enumerators ensured that participants fully understood the study, addressing any questions or concerns before proceeding. Only after confirming comprehension and obtaining explicit verbal agreement did the enumerator proceed with the interview. If the caregiver declined, the next eligible household was approached.

### Outcome definitions

The primary outcome was the prevalence of children among three categories: ‘zero-dose penta’, defined as those who did not receive Penta-1 vaccine by their first birthday; ‘never-immunized’, defined as those who did not receive any of the 19 vaccines part of the WHO-recommended Expanded Programme on Immunization (EPI) vaccination schedule in Pakistan; and ‘immunized’. The ‘immunized’ category was further divided into ‘age-appropriate immunized’, referring to children who had received all vaccines or the vaccines for which they were age-eligible, and ‘under-immunized’, referring to children who had not received all the vaccines for which they were age-eligible. ‘Zero-dose penta’ children were additionally categorized into ‘covered’, indicating those who received the Penta-1 vaccine after 12 months of age, and ‘uncovered’, referring to children who did not receive the Penta-1 vaccine by 23 months of age.

### Statistical analysis

For summary measures, we presented categorical data using frequencies and percentages, and continuous data using medians and interquartile ranges (IQR). We also documented the percentage of missing entries for each variable. Data analysis was conducted using the survey package (svy) in STATA version 17.0, without applying a finite population correction. We used a similar technique as employed for weight assignment during sample size calculation. To evaluate the association between categorical variables and immunization status, we applied the Pearson chi-square test or Fisher’s exact test, as appropriate. Differences in the median values of continuous variables by zero-dose status were assessed using Somer’s D test, with UCs treated as clusters. For analyses involving multiple responses, we transformed each response option into a dummy variable and performed chi-square tests using svy procedures, with Bonferroni correction applied to the alpha value to adjust for multiple comparisons. To identify predictors of immunization status, we conducted both bivariate and multivariable multinomial logistic regression analyses. We employed a manual, bidirectional stepwise selection process for model selection in the multivariable analysis, with gender locked-in as a covariate. First, a univariable analysis was conducted to evaluate each variable’s predictive power, with those demonstrating a p-value < 0.20 considered for inclusion. The multivariable model started with an intercept-only model, and variables were added stepwise based on statistical significance. At each step, the most significant variable from the univariable analysis was introduced, and its impact was assessed in the presence of existing variables. Conversely, any variable in the model with a p-value > 0.10 was removed. This iterative process of adding and removing variables continued until no further variables met the criteria for inclusion or exclusion, ensuring a refined final model. All statistical tests were two-sided, and a p-value of less than 0.05 was considered statistically significant.

## Results

**Fig 1:**
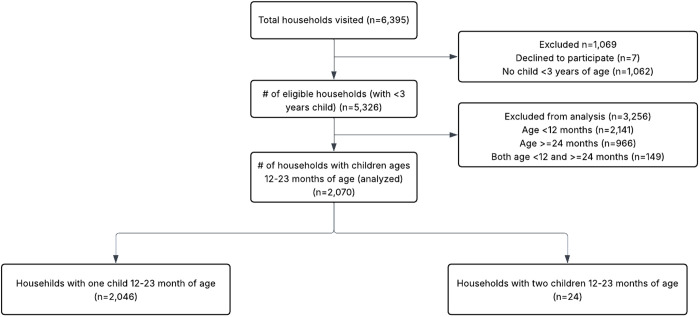
Schema for Households.

A total of 2,070 HHs were included from 6,395 visited (Fig 1).

**Fig 2:**
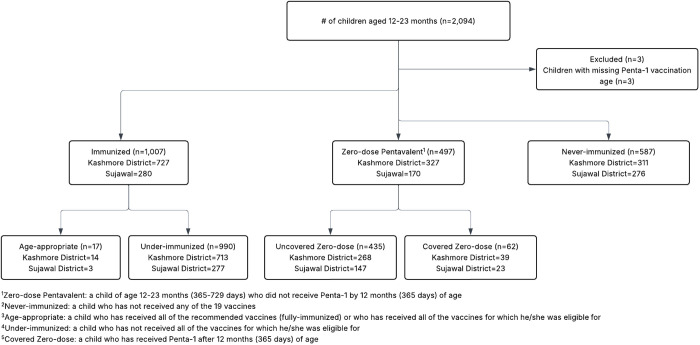
Schema for Children aged 12-23 months (365-729 days).

Among these included HHs, 2,094 children aged 12–23 months were enrolled (Fig 2).

Three children were excluded due to missing data on the Penta-1 vaccine, resulting in a final analysis of 2,091 infants. Of the 2,091 children surveyed, 497 (23.8%) were zero-dose penta, and 587 (28.1%) were never-immunized. Together, these groups constitute 51.9% (1,084/2,091) of the survey population, referred to as ‘true zero-dose’. The remaining 48.1% (1,007/2,091) were immunized, with only 1.7% (17/1,007) achieving age-appropriate vaccination; the majority (98.3%, 990/1,007) were under-immunized. In the zero-dose penta group, only 12.5% (62/497) were covered (achieved coverage of Penta-1 vaccine by 12 months of age), while the remaining 87.5% (435/497) were uncovered (did not achieve coverage of Penta-1 vaccine until 23 months of age).

Nearly half (49.8%, 1,042/2,091) of the enrolled children were girls. The gender disparity (difference in the proportion of boys vs girls) in the zero-dose penta group was negligible (1,004 girls per 1,000 boys). However, for every 100 boys, 117 girls remained never-immunized, and for the immunized group, there were 86 girls vaccinated for every 100 boys.

Table 1 presents the detailed sociodemographic characteristics of the study population. Notably, 95.1% (1,989/2,091) of the enrolled children resided in slum areas. However, 88% of both zero-dose penta and never-immunized children lived within a 5 km radius of EPI centers. A higher proportion of mothers were uneducated compared to fathers (93.8%, 1,961/2,091 vs. 66.9%, 1,400/2,091). Educational attainment was assessed through self-reported data, where participants indicated their highest level of completed education. Fathers were primarily daily wage earners (79.8%, 1,669/2,091), whereas most mothers were homemakers (95.5%, 1,997/2,091). Decisions regarding child health were commonly made jointly by both parents (59.2%, 1,237/2,091 for all health and 46.7%, 977/2,091 for vaccinations specifically). Children who were either immunized (50.0%, 503/1,007) or zero-dose penta (50.5%, 251/497) predominantly spoke Balochi. In contrast, a higher proportion of never-immunized children (50.3%, 295/587) spoke Sindhi. The commute to the nearest healthcare facility, typically a government hospital (62.4%, 867/1,389), took approximately 30 minutes for most participants. Modes of transportation varied: caregivers of immunized (40.8%, 272/666) and zero-dose penta (37.0%, 128/346) children primarily used motorbikes, while those with never-immunized children (35.5%, 134/377) more often used rickshaws. Awareness of Lady Health Worker (LHW) functions was significantly higher among caregivers of immunized children (56.2%, 566/1,007) compared to those of zero-dose penta (43.9%, 218/497) and never-immunized children (41.4%, 243/587). Additionally, HHs that had never been visited by LHWs had a higher proportion of zero-dose penta (55.9%, 278/497) and never-immunized children (55.9%, 328/587).

**Table 1:** Socio-demographic status of 12-23 months children enrolled in the study in Sujawal and Kashmore districts, Sindh by zero-dose status (n = 2,091).

Variables	Children aged 12–23 months (365–729 days)								
	Immunized		Zero-dose		Never immunized		Total		P-value
	n	%	n	%			n	%	
**Total children**	1,007	48.2	497	23.8	587	28.1	2,091	100.0	–
**Area type**									
Slum	940	93.4	477	96.0	572	97.4	1,989	95.1	0.002
Non-slum	67	6.7	20	4.0	15	2.6	102	4.9	
**Child's sex**									
Boy	531	52.7	248	49.9	270	54.3	1,049	50.2	0.03
Girl	476	47.3	249	50.1	317	63.8	1,042	49.8	
**Marital status of parents**									
Currently married	996	98.9	489	98.4	576	98.1	2,061	98.6	0.522
Widow/widower	4	0.4	1	0.2	4	0.7	9	0.4	
Divorced	1	0.1	0	0.0	0	0.0	1	0.1	
Separated	0	0.0	2	0.4	1	0.2	3	0.1	
Missing	6	0.6	5	1.0	6	1.0	17	0.8	
**Father's education**									
0	612	60.8	349	70.2	439	74.8	1,400	67.0	<0.001
1–5	87	8.6	40	8.1	47	8.0	174	8.3	
6–8	27	2.7	9	1.8	19	3.2	55	2.6	
9–10	86	8.5	43	8.7	26	4.4	155	7.4	
>=11	115	11.4	31	6.2	30	5.1	176	8.4	
Missing	80	7.9	25	5.0	26	4.4	131	6.3	
**Mother's education**									
0	924	91.8	473	95.2	564	96.1	1,961	93.8	0.001
1–5	35	3.5	6	1.2	4	0.7	45	2.2	
6–8	13	1.3	0	0.0	3	0.5	16	0.8	
9–10	9	0.9	3	0.6	0	0.0	12	0.6	
>=11	10	1.0	2	0.4	4	0.7	16	0.8	
Missing	16	1.6	13	2.6	12	2.0	2,091)	2.0	
**Parent's literacy**									
Both are illiterate	597	59.3	344	69.2	435	74.1	1,376	65.8	<0.001
Father illiterate only	11	1.1	2	0.4	2	0.3	15	0.7	
Mother illiterate only	273	27.1	110	22.1	112	19.1	495	23.7	
Both are literate	39	3.9	7	1.4	7	1.2	53	2.5	
Missing	87	8.6	34	6.8	31	5.3	152	7.3	
**Father's occupation**									
Daily wager	765	76.0	418	84.1	486	82.8	1,669	79.8	<0.001
Self employed	82	8.1	25	5.0	39	6.6	146	7.0	
Government employee	40	4.0	7	1.4	10	1.7	57	2.7	
Private employee	35	3.5	10	2.0	7	1.2	52	2.5	
Unemployed/ Unable to Work	50	5.0	12	2.4	17	2.9	79	3.8	
Other, specify	28	2.8	21	4.2	20	3.4	69	3.3	
Missing	7	0.7	4	0.8	8	1.4	19	0.9	
**Mother's occupation**									
Daily wager	24	2.4	12	2.4	14	2.4	50	2.4	0.978
Homemaker	966	95.9	476	95.8	555	94.6	1,997	95.5	
Other, specify	8	0.8	3	0.6	6	1.0	17	0.8	
Missing	9	0.9	6	1.2	12	2.0	27	1.3	
**Education of head of household**									
0	528	52.4	303	61.0	354	60.3	1,185	56.7	<0.001
1–5	105	10.4	43	8.7	53	9.0	201	9.6	
6–8	26	2.6	14	2.8	17	2.9	57	2.7	
9–10	72	7.2	35	7.0	23	3.9	130	6.2	
>=11	143	14.2	36	7.2	33	5.6	212	10.1	
Missing	133	13.2	66	13.3	107	18.2	306	14.6	
**Occupation of head of household**									
Daily wager	668	66.3	361	72.6	404	68.8	1,433	68.5	0.002
Self employed	70	7.0	20	4.0	27	4.6	117	5.6	
Government employee	37	3.7	8	1.6	10	1.7	55	2.6	
Private employee	30	3.0	8	1.6	5	0.9	43	2.1	
Unemployed/ Unable to Work	44	4.4	17	3.4	21	3.6	82	3.9	
Other, specify	25	2.5	16	3.2	12	2.0	53	2.5	
Missing	133	13.2	67	13.5	108	18.4	308	14.7	
**Number of children under 3 in a household**									
1	909	90.3	448	90.1	521	88.8	1,878	89.8	0.217
2	91	9.0	49	9.9	65	11.1	205	9.8	
3	7	0.7	0	0.0	1	0.2	8	0.4	
**Family members in a household**									
1–3	186	18.5	94	18.9	95	16.2	375	17.9	0.581
4–6	556	55.2	270	54.3	324	55.2	1,150	55.0	
7–9	217	21.6	98	19.7	131	22.3	446	21.3	
> 9	48	4.8	35	7.0	37	6.3	120	5.7	
**Did the (child's name)’s mother receive antenatal care (ANC) during (child's**									
Yes	598	59.4	265	53.3	301	60.6	1,164	55.7	0.001
No	408	40.5	229	46.1	284	57.1	921	44.1	
Don't know	1	0.1	3	0.6	2	0.4	6	0.3	
**Mother received COVID-19 vaccination**									
Yes	423	42.0	183	36.8	214	43.1	820	39.2	0.409
No	184	18.3	92	18.5	109	21.9	385	18.4	
Don't know	1	0.1	0	0.0	0	0.0	1	0.1	
Missing information	399	39.6	222	44.7	264	53.1	885	42.3	
**Father received COVID-19 vaccination**									
Yes	579	57.5	268	53.9	306	61.6	1,153	55.1	0.19
No	25	2.5	7	1.4	17	3.4	49	2.3	
Don't know	4	0.4	0	0.0	0	0.0	4	0.2	
Missing information	399	39.6	222	44.7	264	53.1	885	42.3	
**Parents received COVID-19 vaccination**									
Both received	399	66.1	177	64.4	203	73.8	779	64.8	0.065
Mother only	23	3.8	6	2.2	11	4.0	40	3.3	
Father only	180	29.8	91	33.1	103	37.5	374	31.1	
None received	2	0.3	1	0.4	6	2.2	9	0.8	
	**Median**	**IQR**	**Median**	**IQR**	**Median**	**IQR**	**Median**	**IQR**	**P-value**
Number of children under 3 in a household	1	45658.0	1	45658.0	1	45658.0	1	45658.0	0.402
Family members in a household	5	45754.0	5	45754.0	5	45754.0	5	45754.0	0.554
Age of father at the time of the survey	30	27-36	30	27-36	31	28-36	30	28-36	0.402
Age of mother at the time of the survey	28	25-32	28	25-32	29	25-33	28	25-32	0.278
Age of head of household at the time of the survey	31	28-38	30	29-38	33	29-38	32	28-38	0.191
**General information**	**n**	**%**	**n**	**%**	**n**	**%**	**n**	**%**	**P-value**
**Has your household been living in this village city since your birth?**									
Yes	993	98.6	484	97.4	575	98.0	2,052	98.1	0.246
No	14	1.4	13	2.6	12	2.0	39	1.9	
**If no, how long ago did your household migrate/start residing in current village/city**									
Less than six months	2	14.3	4	30.8	1	8.3	7	18.0	0.745
6–12 months	2	14.3	1	7.7	3	25.0	6	15.4	
1–2 years	3	21.4	2	15.4	3	25.0	8	20.5	
2–3 years	2	14.3	3	23.1	0	0.0	5	12.8	
3–4 years	2	14.3	1	7.7	2	16.7	5	12.8	
4 years and above	3	21.4	2	15.4	3	25.0	8	20.5	
What was the primary reason of your household migration in this village/city?*									
Better economic opportunity	7	50.0	2	15.4	4	33.3	13	33.3	0.243
Marriage	3	21.4	0	0.0	0	0.0	3	7.7	0.083
Accompany family	2	14.3	5	38.5	6	50.0	13	33.3	0.225
Study of any household member	0	0.0	0	0.0	1	8.3	1	2.6	0.318
Transferred on job	0	0.0	0	0.0	0	0.0	0	0.0	–
Escape from violence/natural disaster	1	7.1	1	7.7	1	8.3	3	7.7	0.963
Others	1	7.1	4	30.8	1	8.3	6	15.4	0.186
Don't know	0	0.0	1	7.7	0	0.0	1	2.6	0.346
**Are there any members of your household who lived here in the past 10 years b**									
Yes	4	0.4	14	2.8	3	0.5	21	1.0	<0.001
No	995	98.8	481	96.8	578	98.5	2,054	98.2	
Don't know	8	0.8	2	0.4	6	1.0	16	0.8	
**What was the main reason that household member(s) moved away?**									
Marriage	4	100.0	11	78.6	1	0.2	16	76.2	0.105
Accompany family	0	0.0	3	21.4	1	0.2	4	19.1	
Transferred on job	0	0.0	0	0.0	1	0.2	1	4.8	
**Mother language**									
Sindhi	375	37.2	186	37.4	295	50.3	856	40.9	<0.001
Balochi	503	50.0	251	50.5	217	37.0	971	46.4	
Others	129	12.8	60	12.1	75	12.8	264	12.6	
**Religion**									
Muslim	998	99.1	490	98.6	580	98.8	2,068	98.9	0.623
Hindu	8	0.8	7	1.4	7	1.2	22	1.1	
Others	1	0.1	0	0.0	0	0.0	1	0.1	
**Type of family structure**									
Nuclear	960	95.3	468	94.2	542	92.3	1,970	94.2	0.068
Joint	47	4.7	29	5.8	45	7.7	121	5.8	
**If joint, share the same kitchen**									
Yes	46	97.9	29	100.0	45	100.0	120	99.2	0.515
No	1	2.1	0	0.0	0	0.0	1	0.8	
**Household income**									
<=10,000	145	14.4	125	25.2	118	20.1	388	18.6	<0.001
10,001–20,000	109	10.8	69	13.9	120	20.4	298	14.3	
20,001–30,000	18	1.8	13	2.6	17	2.9	48	2.3	
30,001–100,000	5	0.5	2	0.4	7	1.2	14	0.7	
>100,000	7	0.7	1	0.2	1	0.2	9	0.4	
Don’t want to disclose	110	10.9	58	11.7	73	12.4	241	11.5	
Don’t know	613	60.9	229	46.1	251	42.8	1,093	52.3	
**What is the structure of your house? (Observe and record)**									
Mud	707	70.2	385	77.5	479	81.6	1,571	75.1	<0.001
Brick and cement	140	13.9	46	9.3	51	8.7	237	11.3	
Mixed	160	15.9	66	13.3	57	9.7	283	13.5	
**Do you own this house or it is rented?**									
Owned	863	85.7	439	88.3	515	87.7	1,817	86.9	0.029
Rented	6	0.6	9	1.8	9	1.5	24	1.2	
Neither owned nor rented	138	13.7	49	9.9	63	10.7	250	12.0	
**Which of these facilities are available in your home?^**									
None	132	13.1	95	19.1	116	19.8	343	16.4	<0.001
Electricity	692	68.7	305	61.4	361	61.5	1,358	65.0	0.002
Radio	4	0.4	0	0.0	3	0.5	7	0.3	0.328
Television	81	8.0	27	5.4	25	4.3	133	6.4	0.020
Telephone/mobile	722	71.7	297	59.8	353	60.1	1,372	65.6	<0.001
Refrigerator	59	5.9	18	3.6	14	2.4	91	4.4	0.003
Air conditioner	17	1.7	2	0.4	2	0.3	21	1.0	0.007
Room cooler	12	1.2	5	1.0	3	0.5	20	1.0	0.417
Washing machine	62	6.2	21	4.2	20	3.4	103	4.9	0.050
Sofa	14	1.4	1	0.2	2	0.3	17	0.8	0.006
Computer	3	0.3	1	0.2	0	0.0	4	0.2	0.353
Sewing machine	44	4.4	16	3.2	17	2.9	77	3.7	0.3391
Camera	2	0.2	1	0.2	0	0.0	3	0.1	0.5327
**Is this household or any household member registered with the Government’s Ehsaas/Benazir Income Support Program?**									
Yes	371	36.8	181	36.4	191	32.5	743	35.5	0.188
No	636	63.2	316	63.6	396	67.5	1,348	64.5	
Which Ehsaas Program, if yes **									
Ehasaas kafaalat	274	73.9	120	38.0	135	34.1	529	71.2	0.166
Ehasaas emergency cash	13	3.5	7	2.2	16	4.0	36	4.9	0.031
Ehasaas nashounuma	4	1.1	4	1.3	1	0.3	9	1.2	0.316
Ehsaas rashan riayat	0	0.0	0	0.0	0	0.0	0	0.0	–
Ehsaas amdan	6	1.6	12	3.8	1	0.3	19	2.6	0.0002
Ehsaas Kafaalat for Special Persons	0	0.0	0	0.0	1	0.3	1	0.1	0.255
Bisp	116	31.3	64	20.3	73	18.4	253	34.1	0.206
Don't know	1	0.3	0	0.0	1	0.3	2	0.3	0.684
**Do any household members have any functional difficulty/disability (seeing, hearing, communication, walking/ climbing, mental/ psychological)?**									
Yes	30	3.0	19	3.8	9	1.5	58	2.8	0.186
No	959	95.2	466	93.8	568	96.8	1,993	95.3	
Don't know	18	1.8	12	2.4	10	1.7	40	1.9	
**Has there been any death of less than 5 years of age child in the household**									
Yes	19	1.9	10	2.0	5	0.9	34	1.6	0.197
No	988	98.1	487	98.0	582	99.2	2,057	98.4	
**What was the age of deceased?**									
0–7 days of life	10	1.0	5	50.0	3	60.0	18	52.9	0.14
8–28 days of life	2	0.2	4	40.0	0	0.0	6	17.7	
29 days – 1 year of life	3	0.3	1	10.0	2	40.0	6	17.7	
1–5 years of life	4	0.4	0	0.0	0	0.0	4	11.8	
**What was the reason of the death? (if 29 days to 5 years of life)**									
Fever	2	28.6	0	0.0	0	0.0	2	20.0	0.276
Injury/accident	1	14.3	1	100.0	1	50.0	3	30.0	
Diarrhea	0	0.0	0	0.0	1	50.0	1	10.0	
Cough/difficulty in breathing	1	14.3	0	0.0	0	0.0	1	10.0	
Weak/underweight	3	42.9	0	0.0	0	0.0	3	30.0	
Others, specify									
**How many times was the mother of [deceased child] pregnant?**									
1	1	8.3	0	0.0	1	33.3	2	8.3	0.243
2	5	41.7	5	55.6	0	0.0	10	41.7	
3	1	8.3	3	33.3	1	33.3	5	20.8	
4	3	25.0	0	0.0	1	33.3	4	16.7	
6	2	16.7	0	0.0	0	0.0	2	8.3	
7	0	0.0	1	11.1	0	0.0	1	4.2	
**If any child in your house becomes sick, who usually makes the decisions about seeking care?**									
Child's mother	116	11.5	67	13.5	94	16.0	277	13.3	<0.001
Child's father	228	22.6	142	28.6	178	30.3	548	26.2	
Jointly by child's father and mother	656	65.1	279	56.1	302	51.5	1,237	59.2	
Child's grandfather/mother	7	0.7	7	1.4	10	1.7	24	1.2	
Others, specify	0	0.0	2	0.4	3	0.5	5	0.2	
**If any child in your house becomes sick, from where do you seek advice or treatment for the child?^^**									
**Public medical sector**									
Govt.hospital	650	64.6	318	64.0	412	70.2	1,380	66.0	0.036
Rural health center	15	1.5	31	6.2	6	1.0	52	2.5	<0.001
Basic health unit	111	11.0	75	15.1	105	17.9	291	13.9	0.0002
Lady Health worker	0	0.0	0	0.0	0	0.0	0	0.0	–
Otherpublic sector	8	0.8	2	0.4	4	0.7	14	0.7	0.674
**Private medical sector**									
Private hospital	450	44.7	181	36.4	206	35.1	837	40.0	0.0001
Private clinic	93	9.2	40	8.1	65	11.1	198	9.5	0.229
Chemist	0	0.0	0	0.0	1	0.2	1	0.1	0.306
Homeopath	78	7.8	39	7.9	87	14.8	204	9.8	<0.001
Dispenser/compounder	1	0.1	0	0.0	0	0.0	1	0.1	0.581
Others	0	0.0	0	0.0	1	0.2	1	0.1	0.247
**Who usually makes the decisions about children's vaccination in the house?**									
Child's mother	220	21.9	99	19.9	158	26.9	477	22.8	<0.001
Child's father	242	24.0	151	30.4	196	33.4	589	28.2	
Jointly by child's father and mother	538	53.4	239	48.1	200	34.1	977	46.7	
Child's grandfather/mother	6	0.6	5	1.0	8	1.4	19	0.9	
Others, specify	1	0.1	3	0.6	25	4.3	29	1.4	
**Is there a health facility near your house?**									
Yes	666	66.1	346	69.6	377	64.2	1,389	66.4	0.147
No	336	33.4	148	29.8	203	34.6	687	32.9	
Don't know	5	0.5	3	0.6	7	1.2	15	0.7	
**What is the type of this nearest health facility?**									
Government hospital	440	66.1	217	62.7	210	55.7	867	62.4	<0.001
Rural health centre	13	2.0	16	4.6	4	1.1	33	2.4	
Basic health unit	170	25.5	98	28.3	135	35.8	403	29.0	
Private hospital	36	5.4	9	2.6	26	6.9	71	5.1	
Private clinic	4	0.6	4	1.2	0	0.0	8	0.6	
Others, specify	3	0.5	2	0.6	2	0.5	7	0.5	
**How do you commute to the nearest health facility?**									
Walk	176	26.4	89	25.7	92	24.4	357	25.7	0.002
Rickshaw	179	26.9	107	30.9	134	35.5	420	30.2	
Public bus	10	1.5	5	1.5	13	3.5	28	2.0	
Taxi	14	2.1	12	3.5	17	4.5	43	3.1	
Private car	6	0.9	1	0.3	8	2.1	15	1.1	
Motorbike	272	40.8	128	37.0	110	29.2	510	36.7	
Others, specify	9	1.4	4	1.2	3	0.8	16	1.2	
**How much time does it take for you to commute to the nearest health facility**									
	**Median**	**IQR**	**Median**	**IQR**			**Median**	**IQR**	
Median (IQR)	30	15-30	30	20-35	30	20-35	30	20-30	0.006
**Is childhood immunization / vaccination service available at your nearest health facility?**									
Yes	569	56.5	283	81.8	283	48.2	1,135	81.7	<0.001
No	74	7.4	48	13.9	30	5.1	152	10.9	
Don't know	23	2.3	15	4.3	64	10.9	102	7.3	
**Do you avail the childhood immunization services available at your nearest health facility?**									
Yes	566	56.2	273	96.5	195	68.9	1,034	91.1	<0.001
No	3	0.3	10	3.5	88	31.1	101	8.9	
**Why do you not avail the childhood immunization services at your nearest health facility?#**									
Health facility is at distant	0	0.0	3	30.0	20	22.7	23	22.8	<0.001
Office hours coincides with centers operational	0	0.0	0	0.0	1	1.1	1	1.0	0.306
Long waiting time	0	0.0	1	10.0	3	3.4	4	4.0	0.101
Unavailability of vaccines	1	33.3	2	20.0	6	6.8	9	8.9	0.065
Unavailability of vaccination staff	0	0.0	1	10.0	11	12.5	12	11.9	<0.001
Poor attitude of facility staff	0	0.0	1	10.0	1	1.1	2	2.0	0.377
Others	2	66.7	4	40.0	50	56.8	56	55.5	<0.001
**Has a vaccinator ever visited your household?**									
Yes	275	27.3	129	26.0	91	15.5	495	23.7	<0.001
No	679	67.4	348	70.0	447	76.2	1,474	70.5	
Don't know	53	5.3	20	4.0	49	8.4	122	5.8	
**When was the last time a vaccinator visited your house?**									
	**Median**	**IQR**	**Median**	**IQR**	**Median**	**IQR**	**Median**	**IQR**	
Median (IQR)	60	30-90	65	30-90	90	60-150	60	30-120	0.002
**Are you aware of the LHW functioning in your area?**									
Yes	566	56.2	218	43.9	243	41.4	1,027	49.1	<0.001
No	363	36.1	240	48.3	275	46.9	878	42.0	
Don't know	78	7.8	39	7.9	69	11.8	186	8.9	
**Has an LHW ever visited your house?**									
Yes	530	52.6	198	39.8	226	38.5	954	45.6	<0.001
No	429	42.6	278	55.9	328	55.9	1,035	49.5	
Don't know	48	4.8	21	4.2	33	5.6	102	4.9	
**When was the last time your household was visited by an LHW?**									
	**Median**	**IQR**	**Median**	**IQR**			**Median**	**IQR**	
Median (IQR)	30	22251.0	30	15-60	30	14-60	30	13-60	0.249
**Has an LHW ever provided information or counselled you on childhood immunization?**									
Yes	285	53.8	116	58.6	113	50.0	514	53.9	0.447
No	243	45.9	81	40.9	111	49.1	435	45.6	
Don't know	2	0.4	1	0.5	2	0.9	5	0.5	

Zero-dose child: a child of age 12–23 months who did not receive Penta-1 by 12 months (365 days) of age.

3 Children with missing Penta-1 vaccination age were excluded from the analysis.

Alpha value: *0.00625; ^0.0038; ** 0.007; ^^ 0.005 and # 0.007.

Bivariate multinomial logistic regression analysis identified six factors associated with both zero-dose penta and never-immunized cohorts. Children residing in slum areas had a significantly higher risk, 1.67 times for being zero-dose penta (RRR = 1.67; 95% CI:1.00–2.79; p < 0.050) and 2.57 times for never being immunized (RRR = 2.57; 95% CI:1.45–4.54; p < 0.001). 

Parental education was a strong determinant of immunization status. Children with uneducated fathers had a two-folded increased risk of being both zero-dose penta and nearly three times the risk of never immunized (zero-dose penta: RRR = 2.15; 95% CI:1.41–3.27; p < 0.001; never-immunized: RRR = 2.69; 95% CI:1.76–4.11; p < 0.001). This risk was further amplified when both parents were illiterate, increasing the likelihood of zero-dose penta by 3.03 times (zero-dose penta: RRR = 3.03; 95% CI:1.33–6.91; p < 0.008) and never-immunized status by 4.21 times (RRR = 4.21; 95% CI:1.84–9.60; p < 0.001). 

Antenatal care (ANC) was another critical factor. Lack of ANC was associated with a 1.34-fold increased risk of being zero-dose penta (RRR = 1.34; 95% CI:1.07–1.67; p < 0.009) and a 1.44-fold higher likelihood of never being immunized (RRR = 1.44; 95% CI:1.17–1.77; p < 0.001). 

Community-level factors also played a significant role. Caregivers unaware of local LHWs had a higher risk of having zero-dose penta (RRR = 1.78; 95% CI:1.42–2.24; p < 0.001) or never-immunized children (RRR = 1.69; 95% CI:1.36–2.11; p < 0.001). Similarly, households never visited by an LHW had a 1.78 times increased risk of being zero-dose penta (RRR = 1.78; 95% CI:1.42–2.24; p < 0.001) and a 1.71 times increased risk of never being immunized (RRR = 1.71; 95% CI:1.38–2.12; p < 0.001) (Table 2).

**Table 2:** Factors associated with zero-dose and never-immunized children among 12-23 months children enrolled in Zero-dose survey in Sujawal and Kashmore districts, Sindh (n = 2,091).

Variables	Bivariate multinomial logistic regression analysis								Multivariable multinomial logistic regression analysis (n = 552)							
	Zerodose				Never immunized				Zerodose				Never immunized			
	RRR	P-value	95% CI		RRR	P-value	95% CI		RRR	P-value	95% CI		RRR	P-value	95% CI	
**Area type**																
Slum	1.67	0.05	1	2.79	2.57	<0.001	1.45	4.54								
Non-Slum																
**Child's sex**																
Boy	Ref				Ref											
Girl	1.11	0.328	0.9	1.39	1.32	0.008	1.08	1.63	1.5	0.07	0.97	2.33	1.43	0.114	0.92	2.22
**Marital status**																
Currently Married	0.88	0.877	0.17	4.66	0.58	0.4	0.17	2.06								
Widow/widower/Divorce/separate	Ref				Ref											
**Father's education**																
0	2.15	<0.001	1.41	3.27	2.69	<0.001	1.76	4.11								
1–5	1.52	0.142	0.87	2.67	1.84	0.029	1.06	3.17								
6–8	1.25	0.617	0.53	2.95	2.48	0.012	1.22	5.05								
9–10	1.8	0.035	1.04	3.1	1.07	0.839	0.58	1.96								
>=11																
**Mother's education**																
0	2.67	0.212	0.57	12.49	1.41	0.563	0.44	4.59								
1-5	0.88	0.891	0.15	5.19	0.24	0.075	0.05	1.16								
6-8					0.51	0.441	0.09	2.86								
9-10	1.89	0.537	0.25	14.26												
>=11	Ref				Ref											
**Parent's literacy**									Ref				Ref			
Both are illiterate	3.03	0.008	1.33	6.91	4.21	0.001	1.84	9.6	3.85	0.052	0.99	23.23	1.82	0.132	0.74	10.09
Father illiterate only	0.82	0.818	0.15	4.53	1.12	0.9	0.2	6.32	2.45	0.471	0.25	19.47	0.56	0.533	0.05	4.83
Mother illiterate only	1.98	0.113	0.85	4.59	2.22	0.064	0.95	5.18	3.18	0.100	0.77	19.41	1.01	0.565	0.39	5.68
Both are literate	Ref				Ref											
**Father's occupation**																
Daily Wager	Ref				Ref											
Self Employed	0.56	0.017	0.34	0.9	0.71	0.098	0.47	1.06								
Government Employee	0.34	0.009	0.15	0.77	0.36	0.005	0.18	0.73								
Private Employee	0.53	0.082	0.26	1.08	0.28	0.003	0.12	0.64								
Unemployed/ Unable to Work	0.45	0.014	0.23	0.85	0.53	0.036	0.29	0.96								
Other, specify	1.33	0.351	0.73	2.4	1.04	0.886	0.58	1.88								
**Mother's occupation**																
Daily Wager	Ref				Ref											
Homemaker	1	0.997	0.49	2.03	0.96	0.893	0.49	1.87								
Other, specify	0.87	0.852	0.19	3.9	1.27	0.708	0.36	4.48								
**Family members in a household**																
3-Jan	Ref				Ref											
6-Apr	0.94	0.698	0.71	1.26	1.12	0.451	0.84	1.49								
9-Jul	0.89	0.505	0.63	1.26	1.18	0.324	0.85	1.65								
> 9	1.34	0.259	0.8	2.25	1.47	0.148	0.87	2.46								
**Did the child's mother receive antenatal care (ANC) during pregnancy?**																
Yes	Ref				Ref											
No	1.34	0.009	1.07	1.67	1.44	0.001	1.17	1.77	1.68	0.035	1.04	2.72	2.07	0.005	1.25	3.45
**Did child's mother receive postnatal care after child's birth?**																
Yes	Ref				Ref											
No	1.26	0.093	0.96	1.64	1.38	0.015	1.07	1.79								
**Mother received COVID-19 vaccination**																
Yes	Ref				Ref											
No	1.18	0.309	0.86	1.61	1.19	0.248	0.89	1.59								
**Father received COVID-19 vaccination**																
Yes	Ref				Ref											
No	0.55	0.176	0.23	1.31	1.29	0.44	0.67	2.49								
**Parents received COVID-19 vaccination**																
Both received	Ref				Ref											
Mother only	0.54	0.19	0.21	1.36	0.93	0.856	0.44	1.97								
Father only	1.16	0.37	0.84	1.59	1.14	0.382	0.85	1.54								
None received	1.18	0.894	0.11	13.2	7.26	0.02	1.37	38.55								
**Poverty score**	0.99	0.238	0.98	1.01	0.98	0.013	0.97	1								
**Age of father at the time of survey**	0.99	0.451	0.98	1.01	1.01	0.273	0.99	1.02								
**Age of mother at the time of survey**	1	0.565	0.98	1.01	1.01	0.275	0.99	1.03								
**Age of head of household at the time of survey**	1	0.622	0.98	1.01	1.01	0.029	1	1.03								
**Number of children under 3 in a household**	0.94	0.753	0.65	1.36	1.06	0.729	0.76	1.48								
**General information**																
**Has your household been living in this village city since your birth?**																
Yes	Ref				Ref											
No	1.93	0.116	0.85	4.39	1.59	0.246	0.73	3.48								
**Mother language**																
Sindhi	Ref				Ref				Ref				Ref			
Balochi	1.08	0.525	0.85	1.36	0.59	<0.001	0.47	0.74	1.34	0.334	0.74	2.41	0.34	0.005	0.16	0.72
Others	0.99	0.959	0.69	1.42	0.77	0.124	0.56	1.07	1.29	0.445	0.67	2.47	0.42	0.036	0.19	0.94
**Religion**																
Muslim	Ref				Ref											
Hindu	1.82	0.25	0.66	5.04	1.6	0.368	0.58	4.44								
Others	–	–	–	–	–	–	–	–								
**Type of family structure**																
Nuclear	Ref				Ref				Ref				Ref			
Joint	1.29	0.305	0.79	2.1	1.67	0.019	1.09	2.58	0.96	0.946	0.3	3.07	2.73	0.057	0.97	7.71
**Household income**																
<=10,000	Ref				Ref								Ref			
10,001–20,000	0.73	0.116	0.49	1.08	1.4	0.068	0.98	2								
20,001–30,000	0.81	0.599	0.36	1.81	1.14	0.73	0.55	2.37								
30,001–100,000	0.49	0.412	0.09	2.68	1.75	0.345	0.55	5.63								
>100,000	0.17	0.096	0.02	1.37	0.19	0.124	0.02	1.57								
**What is the structure of your house? (Observe and record)**																
Mud	1.31	0.089	0.96	1.8	1.88	<0.001	1.36	2.6								
Brick and cement	0.8	0.319	0.51	1.24	0.96	0.86	0.61	1.5								
Mixed	Ref				Ref											
**Do you own this house or it is rented?**																
Owned	Ref				Ref											
Rented	3	0.038	1.06	8.5	2.67	0.064	0.94	7.56								
Neither owned nor rented	0.7	0.046	0.49	0.99	0.78	0.131	0.56	1.08								
**Is this household or any household member registered with the Government’s Ehsaas/Benazir Income Support Program?**																
Yes	Ref				Ref											
No	1.05	0.681	0.84	1.32	1.23	0.068	0.98	1.53								
**Do any household members have any functional difficulty/disability (seeing, hearing, communication, walking/ climbing, mental/ psychological)?**																
Yes	1.16	0.711	0.53	2.51	0.45	0.116	0.16	1.22								
No	Ref				ref											
**Has there been any death of less than 5 years of age child in the household**																
Yes	1.16	0.711	0.53	2.51	0.45	0.116	0.16	1.22								
No	Ref				Ref											
**If any child in your house becomes sick, who usually makes the decisions about it**																
child's mother	Ref				Ref				Ref							
child's father	1.07	0.738	0.74	1.54	0.9	0.534	0.64	1.26	0.27	0.066	0.07	1.09	0.18	0.014	0.05	0.71
jointly by child's father and mother	0.74	0.072	0.53	1.03	0.54	<0.001	0.39	0.73	0.76	0.556	0.31	1.88	0.51	0.215	0.18	1.47
child's grandfather/mother or others	2.33	0.137	0.76	7.14	2.35	0.081	0.9	6.13	0.64	0.717	0.05	7.37	0.26	0.297	0.02	3.26
**Who usually makes the decisions about children's vaccination in the house?**																
child's mother	Red				Ref				Ref				Ref			
child's father	1.35	0.065	0.98	1.85	1.06	0.685	0.8	1.4	3.15	0.076	0.89	11.18	6.76	0.002	1.98	23.07
jointly by child's father and mother	0.99	0.927	0.74	1.31	0.5	<0.001	0.38	0.65	1.15	0.729	0.52	2.55	0.7	0.457	0.28	1.79
child's grandfather/mother and others	2.53	0.099	0.84	7.62	7.58	<0.001	3.24	17.69	2.62	0.505	0.15	44.54	23.27	0.020	1.65	327.65
**Is there a health facility near your house?**																
Yes	Ref				Ref											
No	0.87	0.229	0.68	1.1	1.16	0.173	0.94	1.44								
**Has a vaccinator ever visited your household?**																
Yes	Ref				Ref				Ref				Ref			
No	1.11	0.4	0.87	1.43	1.95	<0.001	1.49	2.55	1.25	0.349	0.78	2.01	4.44	<0.001	2.68	7.36
**Are you aware of the LHW functioning in your area?**																
Yes	Ref				Ref				Ref				Ref			
No	1.78	<0.001	1.42	2.24	1.69	<0.001	1.36	2.11								
**Has an LHW ever visited your house?**																
Yes	Ref				Ref				Ref				Ref			
No	1.78	<0.001	1.42	2.24	1.71	<0.001	1.38	2.12	2.55	0.009	1.26	5.16	1.14	0.684	0.6	2.2

In the multivariable multinomial logistic regression model, lack of ANC remained the only significant predictor for both zero-dose penta and never-immunized status (zero-dose penta: RRR = 1.68; 95% CI:1.04–2.72; p < 0.035; never-immunized: RRR = 2.07; 95% CI:1.25–3.45; p < 0.005). Among zero-dose penta children, additional significant factors included parental illiteracy (RRR = 3.85; 95% CI:0.99–23.23; p < 0.052) and the absence of LHW visits (RRR = 2.55; 95% CI:1.26–5.16; p < 0.009).

For the never-immunized group, key determinants included: mother tongue: Balochi-speaking children were 66% less likely to be never-immunized compared to Sindhi-speaking children (RRR = 0.34; 95% CI:0.16–0.72; p < 0.005), family structure: children in joint families had a higher risk compared to those in nuclear families (RRR = 2.73; 95% CI:0.97–7.71; p < 0.057), decision-making authority: when fathers were the sole decision-makers for general health, the risk of a child never being immunized increased by 6.76 times (RRR = 6.76; 95% CI:1.98–23.07; p < 0.002). However, paternal involvement in general health decisions reduced the risk (RRR = 0.18; 95% CI:0.05–0.71; p < 0.014) and vaccinator visits: households that had never been visited by a vaccinator had a significantly higher risk (RRR = 4.44; 95% CI:2.68–7.36; p < 0.001) (Table 2).

## Discussion

We found that 23.8% of children aged 12–23 months in two low-immunization coverage districts of Sindh, Pakistan, had received zero doses of penta vaccine, and 28.1% were never-immunized. The absence of ANC during pregnancy was significantly associated with an increased risk of both zero-dose pentavalent vaccination and never-immunization, with children being 68% more likely to be zero-dose and twice as likely to be never-immunized. Furthermore, we observed that engagement with frontline health workers (FHWs) was a critical determinant of immunization status. HHs not visited by LHWs experienced a 2.55-fold increase in the prevalence of zero-dose penta children, and the likelihood of children being never-immunized was 4.44 times higher in HHs without vaccinator visits. These findings underscore the importance of strengthening ANC and community-based outreach programs to improve childhood immunization coverage in this region.

Our study uniquely captures the on-the-ground reality of true zero-dose children. Over half (51.9%) of the studied population were classified as true zero-dose, including both zero-dose penta and never-immunized children. This contrasts with prior research that predominantly relies on existing data sources, such as Electronic Immunization Registries (EIRs) and Demographic Health Surveys (DHS), which often mask the extent of children who have yet to make contact with the health system. For example, our observed proportion of children not receiving Penta-1 by their first birthday (23.8%) surpasses the estimate reported in the Pakistan Demographic Health Survey (PDHS) (19.4%), a discrepancy likely stemming from our targeted approach focusing on specific, often marginalized populations. Furthermore, our findings reveal a significantly higher prevalence of zero-dose penta children compared to the Government’s EIR, which reported only one in ten children as zero-dose. This disparity underscores the limitations of relying solely on routine data, which may underestimate the true burden of under-immunization. Notably, the prevalence of zero-dose penta children in our survey exceeds that in other Low and Middle-Income Countries (LMICs), including Bangladesh (1.5%), Nepal (11.1%), and Iraq (14.1%) [27]. While Bangladesh has achieved high immunization coverage through robust community health programs and effective use of digital health tools, Pakistan faces challenges related to security and access in remote regions. Despite Nepal’s demographic similarities to Pakistan, its lower zero-dose pentavalent prevalence highlights the effectiveness of its community health worker network and targeted campaigns. In contrast, Iraq’s conflict and displacement have severely disrupted immunization services, while Pakistan’s challenges stem more from systemic weaknesses within its routine program.

The fact that children in HHs not visited by LHWs were significantly more likely to be zero-dose suggests that the program’s reach is limited. This could be due to factors such as insufficient LHW staffing, inadequate training, or challenges in accessing remote communities. The high number of never immunized children points to the fact that parents are not being adequately counseled on the importance of vaccination, and points to a deficit in the LHW program. These children, effectively left behind, represent Pakistan’s precarious position in the global immunization landscape and its significant challenge in meeting IA 2030 targets. The high prevalence of true zero-dose children indicates that current immunization strategies are failing to reach the most vulnerable populations, necessitating a shift towards more proactive and community-centered approaches. The results suggest that the current electronic immunization registry, while helpful, is not capturing the full picture of the children who have not yet contacted the health system. Therefore, more active case finding and community-level data collection are required.

The notable gender disparity observed in the never-immunized and immunized cohorts suggests potential gender-specific barriers to immunization access. In this context, gender disparities in childhood immunization are likely influenced by deeply embedded societal norms and systemic barriers. In patriarchal communities, healthcare decisions often require male approval, potentially limiting mothers’ autonomy and restricting girls’ access to vaccines [28]. Economic constraints may further exacerbate this inequity, with financially strained HHs potentially prioritizing boys’ healthcare. Structural and logistical challenges, such as conservative norms and safety concerns that discourage families from taking girls to vaccination centers, and a shortage of female health workers limiting community outreach where male providers face restrictions, could also contribute to gender-based disparities in immunization coverage [29]. Misinformation and religious misconceptions, including perceiving vaccines as unnecessary for girls or associating vaccines with sterility myths, may fuel hesitancy [30]. Limited parental education could further reinforce these disparities, perpetuating inequitable immunization coverage [12].

EIRs, while capturing a large proportion, often exceeding 90.0%, of the target population, primarily reflect children already engaged with healthcare services, thus underestimating those never immunized or lost to follow-up, a limitation observed in similar settings utilizing routine health information systems [8]. For instance, studies in Sub-Saharan Africa have noted that relying solely on facility-based data overlooks marginalized populations who rarely access formal healthcare. In contrast, our door-to-door HH survey provides a more comprehensive assessment, capturing both zero-dose pentavalent children and those entirely missed by the health system, mirroring the methodology used in Demographic and Health Surveys (DHS) which are considered the gold standard for population-based health data. This community-based approach, similar to those advocated by the WHO’s REACH initiative, delivers a detailed and accurate depiction of the immunization landscape, enabling precise identification of at-risk populations. Consequently, this study enhances the accuracy and inclusivity of immunization coverage assessments, addressing a critical gap in accurately identifying children missed by EIRs.

Children born to mothers who did not receive ANC faced a significantly higher risk of missing vaccinations, with a 68.0% increased likelihood of not receiving the Penta-1 vaccine and a two-fold increase in the probability of being entirely unvaccinated, compared to children whose mothers accessed ANC. These findings align with previous research; a Pakistani study reported a 40–60% higher likelihood of full vaccination among children whose mothers had 3–4 ANC visits compared to those with fewer visits [31], a finding consistent with studies across LMICs. For example, studies in Nigeria [32] and Myanmar demonstrated an increased likelihood of full vaccination among children of mothers who attended at least four ANC sessions, highlighting the consistent impact of ANC on improving childhood immunization coverage across diverse settings. These findings underscore the critical role of ANC in promoting child immunization, likely due to the anticipatory guidance and health education provided during these visits, which empowers mothers to make informed decisions about their children’s health.

Our analysis indicates a significant association between missed vaccinations and marginalized communities residing in urban slums, characterized by limited access to basic healthcare facilities, a phenomenon observed globally in rapidly urbanizing settings [33]. This observation is consistent with findings from previous studies. Research conducted in informal settlements of Nairobi, Kenya, has demonstrated a high prevalence of zero-dose children, highlighting the challenges faced by populations lacking formal residency and health services [34]. Similarly, a UNICEF report on India’s urban slums revealed elevated rates of under-immunization [35], a trend mirrored in other South Asian megacities where rapid urbanization outpaces infrastructure development. Furthermore, studies in Latin American cities, such as Rio de Janeiro, Brazil, have shown that social exclusion and limited access to outreach programs contribute to low immunization coverage among slum-dwelling children. These results suggest that socioeconomic marginalization, exacerbated by urban poverty and inadequate healthcare infrastructure, critically impacts immunization status, rendering children in these communities disproportionately vulnerable to being excluded from vaccination programs [36]. This highlights the urgent need for targeted interventions that address the specific challenges faced by urban slum populations, recognizing the diverse contexts within which marginalization occurs.

A significant association was observed between HHs not covered by FHWs, including LHWs and vaccinators, and the increased risk of children remaining zero-dose pentavalent or never-immunized. Specifically, children in HHs without LHW or vaccinator visits had a 14% increased risk of being zero-dose pentavalent and a 4.44-fold increased risk of being never-immunized. These findings underscore the critical role of FHW engagement in preventing under-immunization, a role consistently highlighted across diverse settings. For example, studies in Sub-Saharan Africa have demonstrated that community health worker-led interventions significantly improve vaccine uptake, particularly in remote and underserved populations. The substantially higher risk for never-immunized children highlights the necessity of strengthening outreach efforts in areas with a high prevalence of zero-dose and never-immunized cases, a challenge also observed in conflict-affected regions where access to routine immunization services is severely limited. LHWs, as trusted community figures, can effectively address vaccine hesitancy, educate families on the benefits of immunization, and dispel myths surrounding vaccines, a crucial function in settings with low health literacy. However, studies have demonstrated that the effectiveness of LHWs in Pakistan is compromised by infrequent and substandard HH visits, posing significant challenges to meeting global immunization targets, a finding echoed in evaluations of similar programs in other South Asian countries. Therefore, policymakers should prioritize quality and supervision to optimize this critical community resource, ensuring that FHWs are adequately trained and supported. Our findings are consistent with previous research demonstrating the effectiveness of FHW-led interventions in improving childhood immunization rates. For example, a study in rural Bangladesh demonstrated that communities with active FHW outreach programs achieved higher vaccination coverage compared to those with limited outreach [37], a result supported by similar studies in Nepal [38] and Ethiopia [39].

Our study revealed a significant association between ethnic and linguistic disparities and childhood immunization rates, indicating that marginalized groups experience distinct barriers to vaccine access. Specifically, children from Sindhi-speaking HHs were more likely to be never-immunized than those from Balochi-speaking HHs. This disparity reflects broader issues of access and equity within the healthcare system, potentially rooted in cultural differences and varying degrees of social integration. In the local context, distinct cultural practices and communication preferences may influence health-seeking behaviors and the acceptance of immunization services. For instance, Sindhi-speaking communities usually have a more hierarchical social structure, where healthcare decisions are often deferred to elders or male family members, potentially leading to delays or refusals of immunization for female children. Additionally, the prevalence of traditional healing practices within these communities may influence perceptions of modern medicine and vaccine efficacy. Conversely, Balochi-speaking communities, often characterized by a more nomadic or semi-nomadic lifestyle, might face challenges related to access due to geographical remoteness and limited infrastructure. However, they might also demonstrate a stronger reliance on community-based support networks, which could facilitate the dissemination of health information and promote immunization uptake. Furthermore, differing levels of literacy and access to culturally appropriate health information across these linguistic groups could contribute to the observed disparities. Research from developed countries has consistently demonstrated variations in vaccination coverage among ethnic groups, often linked to systemic obstacles such as inadequate access to healthcare services and a lack of culturally appropriate health information. However, the specific influence of ethnicity on zero-dose pentavalent prevalence in Pakistan remains under-explored, highlighting a critical area for further research. A deeper understanding of the cultural and systemic factors affecting marginalized communities is essential for developing targeted interventions to reduce zero-dose pentavalent rates and improve overall health equity.

To achieve global immunization targets and uphold the commitment of ‘Leave no one behind’, policymakers must prioritize understanding the sociodemographic determinants of true zero-dose children. Our study underscores the critical role of ANC programs in reducing zero-dose prevalence. Therefore, we recommend targeted interventions to strengthen and expand access to ANC services, particularly in marginalized communities. Specifically, ANC programs should integrate culturally sensitive immunization counseling, leveraging the established link between ANC attendance and vaccine uptake. Furthermore, given the significant influence of parental education, especially maternal education, on childhood immunization rates, we urge policymakers to invest in targeted educational interventions for girls and women, recognizing the broader public health benefits beyond immunization. To enhance the identification and outreach to true zero-dose children, we recommend strengthening community-based outreach programs in collaboration with partner organizations. This should include the strategic deployment of mobile immunization vans to geographically isolated clusters with high numbers of unvaccinated children, as well as equipping community health workers (CHWs), LHWs, and vaccinators with targeted training and resources to identify and engage these children in marginalized communities. These frontline workers should be trained in culturally sensitive counseling techniques to address vaccine hesitancy and logistical barriers faced by caregivers. Moving forward, research efforts should transcend cross-sectional data collection to explore the complex interplay of factors influencing immunization uptake. We advocate for the integration of qualitative and mixed-methods approaches, including longitudinal studies, to elucidate the barriers faced by marginalized communities and identify causal pathways. Future research should focus on determining the specific cultural and social norms that influence vaccine acceptance in Sindhi-speaking versus Balochi-speaking communities, investigating how intersectional factors, such as gender, socioeconomic status, and geographic location, interact to impact immunization access, and evaluating the most effective community-led interventions for addressing vaccine hesitancy and improving immunization coverage among urban slum populations. These research endeavors will enhance the accuracy and applicability of findings, ultimately informing more effective and targeted public health interventions.

This study, while providing valuable insights into childhood immunization coverage in Sindh, Pakistan, is subject to several limitations. Firstly, the data, collected from a specific subset of the population, may not fully represent the broader demographic, potentially introducing selection bias. Secondly, the reliance on maternal recall for key variables, including vaccination status and ANC attendance, in cases where the date of birth was unknown, introduces the potential for recall bias, a common limitation in HH surveys. While structured questionnaires and cross-verification were employed to minimize this bias, minor inaccuracies may persist. Thirdly, the data collection period, coinciding with the severe floods of 2022, may have impacted caregiver priorities and immunization behaviors, potentially confounding the observed associations. The widespread flooding led to logistical challenges and restricted access to certain communities, necessitating a delay and extension of the data collection period. Furthermore, the cross-sectional design of this study precludes the establishment of causal relationships, and unmeasured confounders, such as socioeconomic status, parental education, and socio-cultural influences, that may have influenced the observed associations. Notably, the observed association between the absence of ANC and increased risk of zero-dose pentavalent and never-immunized children should be interpreted with caution, considering these methodological constraints. Despite these limitations, which are inherent in community-based research, the study’s findings contribute valuable evidence to inform public health interventions. Future research should employ longitudinal and mixed-methods designs to address these limitations, refine data collection methods, and explore causal pathways, thereby enhancing the accuracy and applicability of results in shaping effective immunization strategies.

## Conclusion

We found that one in every two children were true zero-dose (zero-dose penta: 23.8% and never-immunized: 28.1%) in two low-coverage districts of Sindh, Pakistan, revealing significant vaccination gaps. Limited access to ANC and inadequate engagement with FHWs emerged as key contributors. Addressing these disparities through improved ANC access, enhanced FHW care and consideration of community outreach efforts is crucial for achieving universal immunization, aligning with the Immunization Agenda 2030’s zero-dose target.

### Highlights

1. Half of the surveyed children were ‘true zero-dose,’ comprising zero-dose penta (23.8%) and never immunized (28.1%) children.
2. Mothers who did not receive ANC during pregnancy were 68% more likely to have children categorized as zero-dose penta, and over twice as likely to have never-immunized children.
3. HHs with no LHW visits were 2.6 times more likely to have zero-dose penta children.
4. HHs with no vaccinator visits had a 4.4 times greater risk of having never-immunized children.
5. Increasing access to ANC and active engagement of FHWs emerge as potential channels to improve vaccination status of zero-dose penta and never-immunized children.

### Evidence before this study

We searched PubMed, Google Scholar, Demographic and Health Surveys, the UNICEF Multiple Indicator Cluster Surveys, and other survey series between 2018 and 2023 for publications in the past 6 years, using search terms “Zero-dose”, “Unvaccinated”, “Household Survey” and “Factors associated with zero-dose children”. We found more than 200 articles focusing on overall coverage and timeliness of routine immunizations in low and middle-income countries including Pakistan. Less than 10 articles evaluated the prevalence of zero-dose children and their predictors in low and middle-income countries including Pakistan. All the articles were based on survey data collection or sampling. Few articles investigated the prevalence of zero-dose children enrolled through a digital immunization registry.

### Added value of this study

To our knowledge, this is the first analysis to investigate the community-based prevalence of zero-dose children among children aged 12–23 months and identify their predictors in low-coverage districts of Sindh, Pakistan through a HH survey. Based on data from 6,395 HHs and 2,091 children, the study aims to estimate the prevalence of zero-dose penta and never-immunized children. Additionally, the research identifies the sociodemographic disparities in each group. The findings explore the factors influencing the likelihood of a child belonging to either the zero-dose penta or never-immunized group, through adjusted regression analysis.

### Implications of all the available evidence

This study identifies a higher prevalence of zero-dose penta and never-immunized children in community settings than reported by the Pakistan Demographic Health Survey (PDHS) and through administrative data. This highlights critical gaps in achieving universal immunization. Our findings emphasize the need for targeted public health interventions, particularly in underserved populations, such as emphasizing the importance of ANC and engagement with FHWs. Future strategies must focus on data-driven methods to address these immunization disparities, supported by collaborative efforts to strengthen health systems across socioeconomic and geographic divides.

## Supporting information

S1 Table Sampling frame for District selection. (DOCX)

S2 Table Sampling frame for UCs Selection. (DOCX)

S3 Table Sample size in different scenario. (DOCX)

S1 File Inclusivity-in-global-research-questionnaire. (DOCX)

## References

[1] UNICEF. The State of the World’s Children 2023: For every child, vaccination. Florence: UNICEF Innocenti – Global Office of Research and Foresight. 2023.

[2] World Health Organization (WHO). WHO UNICEF Immunization Coverage Estimates 2023 revision. 2023.

[3] Osman MA, Waits A, Chien LY. Factors associated with vaccination coverage among 0–59-month-old children: A multilevel analysis of the 2020 Somaliland demographic and health survey. Vaccines. 2024;12(5):509. 38793760 10.3390/vaccines12050509 PMC11125891

[4] Ingle EA, Shrestha P, Seth A, Lalika MS, Azie JI, Patel RC. Interventions to Vaccinate Zero-Dose Children: A Narrative Review and Synthesis. Viruses. 2023;15(10):2092. doi: 10.3390/v15102092 37896868 PMC10612020

[5] GAVI. The Zero-Dose Child: Explained. https://www.gavi.org/vaccineswork/zero-dose-child-explained 2021

[6] Farrenkopf BA, Zhou X, Shet A, Olayinka F, Carr K, Patenaude B, et al. Understanding household-level risk factors for zero dose immunization in 82 low- and middle-income countries. PLoS One. 2023;18(12):e0287459. doi: 10.1371/journal.pone.0287459 38060516 PMC10703331

[7] World Health Organization WHO. Immunization Agenda 2030: A global Strategy to Leave No One Behind. Geneva, Switzerland: World Health Organization. 2020. https://www.who.int/teams/immunization-vaccines-and-biologicals/strategies/ia2030

[8] Saeed R, Hashmi I. Pakistan ranks third globally with the most unvaccinated children: is the impact of parental perception and attitude on immunization an essential contributing factor to an unsuccessful vaccination coverage?. Cureus. 2021;13(11):e19751. doi: 10.7759/cureus.19751 34938628 PMC8684801

[9] Mehmood M, Setayesh H, Siddiqi DA, Siddique M, Iftikhar S, Soundardjee R, et al. Prevalence, geographical distribution and factors associated with pentavalent vaccine zero dose status among children in Sindh, Pakistan: analysis of data from the 2017 and 2018 birth cohorts enrolled in the provincial electronic immunisation registry. BMJ Open. 2022;12(5):e058985. doi: 10.1136/bmjopen-2021-058985 35584879 PMC9119190

[10] Hogan D, Gupta A. Why Reaching Zero-Dose Children Holds the Key to Achieving the Sustainable Development Goals. Vaccines (Basel). 2023;11(4):781. doi: 10.3390/vaccines11040781 37112693 PMC10142906

[11] Butt M, Mohammed R, Butt E, Butt S, Xiang J. Why have immunization efforts in Pakistan failed to achieve global standards of vaccination uptake and infectious disease control?. Risk management and healthcare policy. 2020;:111–24. 32104117 10.2147/RMHP.S211170 PMC7024803

[12] Hakim M, Ali F, Zala Z, Pervaiz A, Afaq S, Haq Z. Prevalence and associated factors of parental refusal rates for routine immunisation: a cross-sectional study in Peshawar, Khyber Pakhtunkhwa, Pakistan-2024. BMC Public Health. 2025;25(1):369. 39881273 10.1186/s12889-025-21388-1 PMC11781007

[13] UNICEF. Gender barriers to health and immunization in East Asia and the Pacific. 2025.

[14] CIA’s misleading inoculation drive led to vaccine decline in Pakistan. EurekAlert. https://www.eurekalert.org/news-releases/529936 2021

[15] EPI Annual Targets. Karachi, Pakistan: Office of the Project Director, Expanded Programme on Immunization (EPI). 2022.

[16] Pakistan Bureau of Statistics (PBS) final results (Census-2017). Pakistan Bureau of Statistics. 2017. https://www.pbs.gov.pk/content/final-results-census-2017

[17] Oxford Poverty & Human Development Initiative (OPHI), UNICEF, Ministry of Planning Development & Special Initiatives Government of Pakistan. Multidimensional Poverty Index Report 2019-20.

[18] Chandir S, Siddiqi DA, Dharma VK, Shah MT, Turab A, Khan MI. Zindagi Mehfooz (safe life) digital immunization registry: Leveraging low-cost technology to improve immunization coverage and timeliness in Pakistan. Iproceedings. 2018;4(2):e11770.

[19] Sindh electronic immunization registry (SEIR) analysis. 2023.

[20] EPI Sindh. Mid-term review for national immunization support project (NISP). 2020.

[21] Soofi SB, Hussain I, Shah MA, Safdar RM, Umer M, Khan A. Third-Party Verification Immunization Coverage Survey (TPVICS)-2021. 2021.

[22] Zaidi S. Sindh health sector strategy 2012–2020. 2012.

[23] Baran KA. Minute Mirror. https://minutemirror.com.pk/sujawal-districts-development-challenges-335095/. 2025

[24] The Sindh Katchi Abadis Act, 1987. 1987.

[25] UNICEF. Report of Profiles of Slums/U underserved Areas of 08 Largest Cities of Pakistan. Unicef. 2020.

[26] Khawar H. Vaccination drive 2021. https://www.dawn.com/news/1657331 Accessed 2023 October 1

[27] Wonodi C, Farrenkopf BA. Defining the Zero Dose Child: A Comparative Analysis of Two Approaches and Their Impact on Assessing the Zero Dose Burden and Vulnerability Profiles across 82 Low- and Middle-Income Countries. Vaccines (Basel). 2023;11(10):1543. doi: 10.3390/vaccines11101543 37896946 PMC10611163

[28] Merten S, Martin Hilber A, Biaggi C, Secula F, Bosch-Capblanch X, Namgyal P, et al. Gender Determinants of Vaccination Status in Children: Evidence from a Meta-Ethnographic Systematic Review. PLoS One. 2015;10(8):e0135222. doi: 10.1371/journal.pone.0135222 26317975 PMC4552892

[29] Davas A, Etiler N. Gender differences in cost-related unmet healthcare needs: a national study in Turkiye. BMC Public Health. 2024;24(1):2413. doi: 10.1186/s12889-024-19878-9 39232689 PMC11375860

[30] The Gurdian. Afghanistan risks polio outbreak as Taliban restricts women from delivering vaccines 2024. Available from: https://www.theguardian.com/global-development/2024/sep/17/taliban-curbs-women-risk-polio-outbreak-vaccination-campaign-health-officials-warn

[31] Noh J-W, Kim Y-M, Akram N, Yoo K-B, Park J, Cheon J, et al. Factors affecting complete and timely childhood immunization coverage in Sindh, Pakistan; A secondary analysis of cross-sectional survey data. PLoS One. 2018;13(10):e0206766. doi: 10.1371/journal.pone.0206766 30379947 PMC6209382

[32] Matthew Ayodele A, Fasasi MI, Rejoice Uche O, Gideon Ikemdinachi N, Henry Ugochukwu U. Factors associated with full childhood vaccination coverage among young mothers in Northern Nigeria. Pan Afr Med J. 2024;47:4. doi: 10.11604/pamj.2024.47.4.37517 38371647 PMC10870161

[33] Khaliq A, Ali S, Zahid A, Lokeesan L, Holmes-Stahlman R, Lassi ZS. A Community-Based Survey Exploring the Determinants of Invalid, Delayed, and Missed Immunization in Children of Urban Slums of Karachi, Pakistan. Arch Clin Pediatr. 2024;1(1):10–22. doi: 10.33696/pediatrics.1.003

[34] Erasmus University Rotterdam. From research to action: How community researchers in Nairobi promote social transformation. 2024. https://www.eur.nl/en/news/research-action-how-community-researchers-nairobi-promote-social-transformation

[35] UNICEF. Building trust to reach zero-dose children in India. https://www.unicef.org/stories/sowc-2023/india-reaching-zero-dose-children 2023. Accessed 2024 July 8

[36] Zimba B, Mpinganjira S, Msosa T, Bickton FM. The urban-poor vaccination: Challenges and strategies in low-and-middle income countries. Hum Vaccin Immunother. 2024;20(1):2295977. doi: 10.1080/21645515.2023.2295977 38166597 PMC10766387

[37] Uddin MJ, Larson CP, Oliveras E, Khan AI, Quaiyum MA, Chandra Saha N. Child immunization coverage in rural hard-to-reach Haor areas of Bangladesh: possible alternative strategies. Asia Pac J Public Health. 2009;21(1):8–18. doi: 10.1177/1010539508327030 19124332

[38] Ogutu E, Ellis AS, Hester KA, Rodriguez K, Sakas Z, Jaishwal C. Success in vaccination programming through community health workers: A case study of Nepal, Senegal, and Zambia. medRxiv. 2023. doi: 10.1101/2023.05.05.23289567 PMC11146414 38569679

[39] Nigatu T, Abraham L, Willems H, Tilaye M, Tiruneh F, Gebru F, et al. The status of immunization program and challenges in Ethiopia: A mixed method study. SAGE Open Med. 2024;12:20503121241237115. doi: 10.1177/20503121241237115 38516641 PMC10956145

