# Supplementary material for: Estimating prevalence and identifying predictors of zero-dose pentavalent and never-immunized children under two years of age in Kashmore and Sujawal Districts of Sindh, Pakistan: An analysis of household survey data
Source: PLoS One. 2025 Aug 26;20(8):e0330281. doi: 10.1371/journal.pone.0330281 (PMC12380306; doi:10.1371/journal.pone.0330281)
Supplement: S1 Table — (DOCX) [file pone.0330281.s001.docx]

# S1 Table: Sampling frame for District selection

| **S. No.** | **District*** | **Penta 1** |
| --- | --- | --- |
| 1 | Sujawal^a^ | 48.1 |
| 2 | Kashmore^a^ | 50.5 |
| 3 | Thatta^a^ | 65.0 |
| 4 | Kambar^a^ | 67.5 |
| 5 | Jacobabad | 70.3 |
| 6 | Sanghar | 75.2 |
| 7 | Ghotki | 79.9 |
| 8 | Sukkur | 80.5 |
| 9 | Matiari | 85.4 |
| 10 | Karachi West | 85.6 |
| 11 | Larkana | 86.3 |
| 12 | Benazirabad | 86.3 |
| 13 | Tando Mohd Khan | 86.4 |
| 14 | Dadu | 87.0 |
| 15 | Jamshoro | 87.0 |
| 16 | Badin | 87.2 |
| 17 | Malir | 87.6 |
| 18 | Hyderabad | 87.8 |
| 19 | Mirpur Khas | 88.0 |
| 20 | Shikarpur | 89.2 |
| 21 | Naushahro Feroze | 89.4 |
| 22 | Tharparkar | 90.0 |
| 23 | Karachi East | 90.8 |
| 24 | Umer Kot | 91.1 |
| 25 | Tando Allahyar | 93.2 |
| 26 | Karachi Central | 93.4 |
| 27 | Khairpur | 94.2 |
| 28 | Korangi | 94.2 |
| 29 | Karachi South | 95.7 |

Pentavalent 1 Coverage reported in Third party verification immunization Coverage Survey 2021, AKU

*In April 2021, District West was divided into two districts: 1) District Kemari and 2) District West

a:10th percentile
