# Supplementary material for: Estimating prevalence and identifying predictors of zero-dose pentavalent and never-immunized children under two years of age in Kashmore and Sujawal Districts of Sindh, Pakistan: An analysis of household survey data
Source: PLoS One. 2025 Aug 26;20(8):e0330281. doi: 10.1371/journal.pone.0330281 (PMC12380306; doi:10.1371/journal.pone.0330281)
Supplement: S2 Table — (DOCX) [file pone.0330281.s002.docx]

# S2 Table: Sampling frame for UCs Selection

| **S. No.** | **Khasmore UCs selection**  **(n=33)** | | **Sujawal UCs selection**  **(n=25)** | |
| --- | --- | --- | --- | --- |
|  | **UCs**** | **Pent a1 Coverage** | **UCs** | **Penta 1 Coverage** |
| 1 | Haibat^a^ | 37.30% | Goongani^a,b^ | 28.40% |
| 2 | Tangwani UC^a,b^ | 38.40% | Jaar^a,b^ | 31.40% |
| 3 | Saifal^a^ | 38.90% | Darya Khan Soho^a^ | 34.80% |
| 4 | Kajili^a^ | 39.50% | Begna Mori^a^ | 35.00% |
| 5 | Duniapur^a^ | 53.10% | Mehar Shah^a,b^ | 37.20% |
| 6 | Daulatpur^a,b^ | 54.00% | Ali Bahar^a^ | 40.40% |
| 7 | Sohlyani^a^ | 54.50% | Doulatpur^a^ | 42.60% |
| 8 | Kashmore Colony^a,b^ | 60.20% | Keenjhar^a,b^ | 47.40% |
| 9 | Kandhkot- Malir^a^ | 62.60% | Gul Muhammad Baran | 51.00% |
| 10 | Zorgarh^a,b^ | 65.90% | Kothi | 51.80% |
| 11 | K S Ali Bilawal^a,b^ | 66.80% | Jhok sharif | 59.00% |
| 12 | Lashari UC | 68.10% | Bello | 60.90% |
| 13 | Jamal | 69.40% | Jango Jalbani | 61.60% |
| 14 | Dari | 71.90% | Ladiyoon | 69.40% |
| 15 | Kumb | 74.20% | Kar Malik | 73.40% |
| 16 | Lalao | 76.60% | Mirpur Bathoro | 80.70% |
| 17 | Cheel | 98.50% | Bajora | 86.10% |
| 18 | Geehalpur | 103.20% | Bannu | 87.30% |
| 19 | Khewali | 112.20% | Laikpur | 92.00% |
| 20 | Gulanpur | 114.10% | Mureed Khoso | 100.40% |
| 21 | Kashmore 2 | 114.70% | Chuhar Jamali | 110.60% |
| 22 | Karampur | 121.90% | Daroo | 125.10% |
| 23 | Akhero | 128.10% | Bachal gugo | 130.90% |
| 24 | Buxapur | 129.10% | Sujawal | 133.60% |
| 25 | Sodhi | 129.20% | Mughal Bean | 145.90% |
| 26 | Badani | 131.10% |  |  |
| 27 | Resaldar | 145.40% |  |  |
| 28 | Guddu | 160.20% |  |  |
| 29 | R B Chacher | 171.10% |  |  |
| 30 | Ghouspur | 186.70% |  |  |
| 31 | Kashmore 1 | 257.60% |  |  |
| 32 | Gulwali | 361.90% |  |  |
| 33 | Kandhkot-4 | 441.60% |  |  |

UCs sorted out in ascending order based on the pentavalent 1 coverage*
*Crude Pentavalent-1 vaccination coverage rates in estimated 2020 surviving annual birth cohort in ZM-EIR in Sujawal, by union councils (UCs) (Jan 1, 2020-Aug 31, 2021)

** In 4 UC there is no EPI center

a: one-third UCs having lowest pentavalent 1 coverage*

b: one-third UCs selected using simple random sampling
