# Supplementary material for: Estimating prevalence and identifying predictors of zero-dose pentavalent and never-immunized children under two years of age in Kashmore and Sujawal Districts of Sindh, Pakistan: An analysis of household survey data
Source: PLoS One. 2025 Aug 26;20(8):e0330281. doi: 10.1371/journal.pone.0330281 (PMC12380306; doi:10.1371/journal.pone.0330281)
Supplement: S3 Table — (DOCX) [file pone.0330281.s003.docx]

# S3 Table: Sample size in different scenario

| **Proportion of stratum h showing characteristic (h1=h2)** | **10%** | **20%** | **30%** | **40%** | **42%** | **50%** | **52%** | **58%** |
| --- | --- | --- | --- | --- | --- | --- | --- | --- |
| **Absolute precision required** | 0.01 | 0.02 | 0.03 | 0.04 | 0.04 | 0.05 | 0.05 | 0.06 |
| **Estimated sample size** | 3,452 | 1536 | 896 | 577 | 531 | 385 | 355 | 279 |
| **Adding design effect for cluster sampling (2)** | 6,904 | 3,072 | 1,792 | 1,154 | 1,062 | 770 | 710 | 558 |
| **Required sample size with 28% refusal rate** | 8,837 | 3,932 | 2,294 | 1,477 | 1,359 | 986 | 909 | 714 |
| **N_HH_ to visit** | 61,860 | 27,525 | 16,056 | 10,340 | 9,516 | 6,899 | 6,362 | 5,000 |
| **Required sample size with 28% refusal rate for Kashmore** | 5,154 | 2,293 | 1,338 | 862 | 793 | 575 | 530 | 417 |
| **Required sample size with 28% refusal rate for Sujawal** | 3,683 | 1,639 | 956 | 616 | 567 | 411 | 379 | 298 |
| **N_HH_ to visit in Kashmore** | 36,080 | 16,054 | 9,365 | 6,031 | 5,550 | 4,024 | 3,710 | 2,916 |
| **N_HH_ to visit in Sujawal** | 25,780 | 11,471 | 6,691 | 4,309 | 3,966 | 2,875 | 2,653 | 2,084 |

Assumptions: level of confidence 95%, absolute precision 10%, number of strata 2, all ages population size of Kashmore 10,90,336 and Sujawal 7,79,062, relative weight for Kashmore 58 and Sujawal 42, Non-response rate 28%.
